# Role of Diffusion-Weighted Imaging in the Evaluation of Post-Treatment Tumor Response in Rectal Carcinoma

**DOI:** 10.7759/cureus.17471

**Published:** 2021-08-26

**Authors:** Pir Abdul Ahad Aziz Qureshi, Javaria Aleem, Nadia Mushtaq, Muhammad Asif Noor, Imran Khalid Niazi, Muhammad Omer Altaf

**Affiliations:** 1 Department of Radiology, Shaukat Khanum Memorial Cancer Hospital and Research Centre, Lahore, PAK; 2 Department of Surgical Oncology, Shaukat Khanum Memorial Cancer Hospital and Research Centre, Lahore, PAK

**Keywords:** rectal cancer, mri rectum, diffusion-weighted imaging (dwi), concurrent chemoradiotherapy, tumor imaging

## Abstract

Introduction

Rectal cancer has become a major cause of mortality worldwide. Imaging has a primary role in staging and assessing the response to therapy. MRI is superior to all other modalities in local staging of the rectal tumor and in predicting tumor response. Pelvic MRI has an undeniable role in the therapeutic management of rectal cancer, particularly for the determination of the circumferential resection margin (CRM), evaluation of sphincter invasion, and assessment of the extramural vascular invasion. Post-chemoradiotherapy (CRT) staging aims at assessing treatment response and choosing methods for further treatment such as surgical resection or extended CRT. MRI with diffusion restriction is a non-invasive and useful tool for assessing the treatment response of locally advanced lower rectal cancer. It will reduce the burden of extensive abdominoperineal resection (APR) surgery in patients.

Objective

The purpose of this study was to determine the role of diffusion-weighted imaging (DWI) in the evaluation of post-treatment tumor response in rectal carcinoma.

Materials and methods

The study was approved by our institutional review board, which waived the requirement for informed consent. The clinical data of all the patients treated for rectal carcinoma at the Shaukat Khanum Memorial Cancer Hospital and Research Centre, Lahore between February 1, 2014, and February 28, 2019, were retrospectively evaluated. The inclusion criteria were as follows: (1) patients with histopathologically proven rectal adenocarcinoma, (2) those who underwent APR before February 2019 at our hospital, and (3) those who underwent MRI including DWI/apparent diffusion coefficient (ADC) imaging before and after CRT. Those patients who had upfront surgery without neoadjuvant CRT and those who did not have MRI imaging with DWI/ADC were excluded from the study.

Results

A total of 200 patients who fulfilled the inclusion criteria were included in this study. Among those, 141 were males and 59 were females. On histology, 110 had moderately differentiated adenocarcinoma, 25 had well-differentiated adenocarcinoma, and 65 had poorly differentiated adenocarcinomas. Overall diagnostic accuracy of DWI MRI sequence was calculated to be 91%, while the sensitivity was 98.09%, specificity was 65.12%, positive predictive value was 91.12%, and negative predictive value was 90.32%.

Conclusion

DWI was proven to be very useful in the post-treatment evaluation of tumor response with very high diagnostic accuracy.

## Introduction

Rectal cancer is becoming a major cause of mortality worldwide. MRI is frequently used as a standard modality for the local staging of rectal cancer and to evaluate the therapy response [1]. Restaging MRI plays a vital role in the evaluation of the response of locally advanced cancer to neoadjuvant chemoradiotherapy (CRT) and helps in making appropriate management choices, such as reduction in the surgical resection in cases of a significant reduction in tumor size, organ-preserving options in cases of near-complete response, or a "wait and watch" policy [2-4]. Currently, MRI with diffusion-weighted imaging (DWI) is being increasingly used for restaging in the post-CRT rectal tumors as it increases the accuracy of residual disease detection [5-8]. In light of this, the purpose of this study was to identify the role of DWI in the evaluation of post-treatment tumor response in rectal carcinoma.

## Materials and methods

Patient selection

The study was approved by our institutional review board, which waived the requirement for informed consent. The clinical data of all the patients treated for rectal carcinoma at the Shaukat Khanum Memorial Cancer Hospital and Research Centre, Lahore between February 1, 2014, and February 28, 2019, were retrospectively evaluated. The inclusion criteria were as follows: (1) patients with histopathologically proven rectal adenocarcinoma, (2) those who underwent abdominoperineal resection (APR) before February 2019 at our hospital, and (3) those who underwent MRI including DWI/apparent diffusion coefficient (ADC) imaging before and after CRT. Patients who had undergone upfront surgery without neoadjuvant CRT and those who did not have MRI with DWI/ADC were excluded from the study. Subsequently, 200 patients who fulfilled the inclusion criteria were included in the study.

MRI technique

All selected patients had undergone MRI pelvis without contrast as baseline workup and after CRT. All pre- and post-CRT imaging were performed on a 1.5 Tesla MRI (Philips Ingenia, Philips Healthcare, Best, Netherlands) and 3.0 Tesla MRI (Siemens Magnetom Vida, Siemens Healthineers AG, Erlangen, Germany) systems. Bowel preparation with purgative and six hours of fasting before scanning time was done. The MRI protocol included the multiparametric MRI sequences with DWI/ADC. A T2-weighted fast spin-echo sequence, a T1-weighted spin-echo sequence, and an oblique axial DWI sequence were acquired. Routine axial, sagittal, and coronal images were also obtained. We used b factor of 0 and 1000 sec/mm^2^ to obtain high b-value DWI images. The total examination time was approximately 30 minutes. The average interval between post-CRT MRI and surgery was eight weeks (range: 1-12 weeks).

Image interpretation

MRI images of all the selected patients were reviewed retrospectively on a picture archiving and communication system workstation monitor by two Radiology fellows (from Body Imaging and PET-CT subspeciality) independently; this was supervised by one Radiology consultant having experience of more than 10 years. The Radiology fellows and Radiology consultants were blinded to the previous MRI reports and surgical and pathological outcomes. The location of the tumor was categorized as the distal rectum and anal canal (within 4.0 cm of the anal verge, the anal verge is the lowest part of the anal canal that starts where the skin ends and the anal mucosa starts), distal rectum (within 4.1-8.0 cm of the anal verge), middle rectum (within 8.1-12.0 cm of the anal verge), proximal rectum (within 12.1-16.0 cm of the anal verge), and whole rectum. On the pre-treatment MRI scan, the tumor stage, T2 tumor signals, and diffusion restriction were identified. Tumor response was determined according to the Response Evaluation Criteria in Solid Tumours (RECIST) guidelines. Complete response (CR) was predefined as the disappearance of measurable tumor volume. The absence of residual disease was also defined as CR. The absence of tumor signals on T2 and DWI was also defined as CR or no residual disease. Partial response (PR) was defined as a 30% reduction in the tumor's longest dimension. Progressive disease (PD) was defined as a 20% increase in the longest dimension. Stable disease was predefined as no response that failed to meet the criteria of CR, PR, and PD.

Statistical analysis

Analysis was done using SPSS Statistics version 25 (IBM, Armonk, NY). We calculated percentages of different responses of tumors to neoadjuvant therapy. Tumor response and pathological response correlation were determined by the Chi-square test. Diagnostic accuracy, sensitivity, and specificity of MRI in determining tumor response were calculated; a 95% confidence interval was used to determine statistical precession, and p-values were calculated. The diagnostic accuracy of the DWI sequence was calculated using the receiver operating characteristic (ROC) curve analysis.

## Results

Patient demographics

A total of 200 patients were included in this study, with an age range of 21-73 years; all of them underwent low APR. Of these 200 patients, 141 were males and 59 were females. The average interval between post-CRT MRI and surgery was eight weeks (range: 1-12 weeks). The location of tumor was categorized as distal rectum and anal canal (n=83), distal rectum (n=41), middle rectum (n=48), proximal rectum (n=16), and whole rectum (n=12). All patients underwent pre-treatment colonoscopy-guided biopsy. On histology, 110 had moderately differentiated adenocarcinoma, 25 had well-differentiated adenocarcinoma, and 65 had poorly differentiated adenocarcinomas. Both the pre-CRT local staging based on MRI and the post-CRT pathologic staging of the cases are summarized in Table 1.

**Table 1:** Radiological and pathological tumor staging of study cases CRT: chemoradiotherapy; APR: abdominoperineal resection; MRI: magnetic resonance imaging

Pre-CRT/APR					
		MRI staging			Total
		T2	T3	T4	
	N0	1	16	0	17
	N1	8	91	9	108
	N2	3	66	6	75
Total		12	173	15	

Post-CRT/APR						
		Pathology staging				Total
		T2	T3	T4a	No residual	
	N0	33	31	0	34	98
	N1	13	25	6	4	48
	N2	4	41	9	0	54
Total		50	97	15	38	200

Evaluation of tumor response

After neoadjuvant CRT, tumor response was evaluated on a post-CRT MRI scan including DWI and ADC sequences. We determined the response on the basis of decrease in the tumor size, signal change on T2WI sequence, presence of necrosis within the tumor, which appears bright on T2WI, and the absence of diffusion restriction on DWI. Some cases showed no measurable disease on the T2 sequence but diffusion restriction was seen on the DWI sequence, and those cases were labeled as PR. We called CR on an MRI scan when no measurable disease was present on the T2 sequence and no diffusion restriction was detected. Post-CRT MRI results were compared with post-APR histopathology results. A total of 200 cases were evaluated. There was a statistically significant correlation between MRI tumor response grading and pathological grading (p=0.000). Out of these 200 cases, 28 (14%) cases showed no residual disease on post-CRT MRI scans. Post-APR histopathology of these cases confirmed the absence of the disease; 15 (7.5%) cases showed residual disease on MRI but they were negative on post-APR histopathology. Of note, 154 (77%) cases showed the presence of residual disease on MRI as well as on post-APR histopathology. Out of these 154 cases, 12 (6%) had SD, 135 (67.5%) showed PR, and seven (3.5%) had PD. Three (1.5%) cases showed absent disease on MRI but post-APR histopathology residual disease was present in them. MRI DWI tumor response was significantly correlated with the pathological response (p=0.000). Overall diagnostic accuracy of DWI MRI sequence was calculated to be 91%; the sensitivity was 98.09%, specificity was 65.12%, positive predictive value was 91.12%, and negative predictive value was 90.32%.

Diagnostic performance of DWI to identify complete response

**Figure 1:**
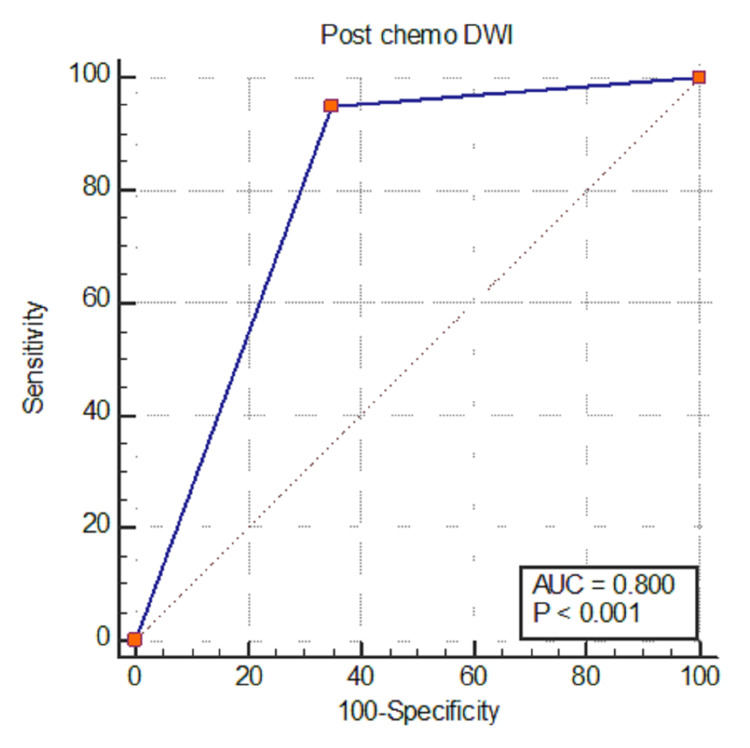
Area under the ROC curve (AUC) DWI: diffusion-weighted imaging; ROC: receiver operating characteristic

On post-neoadjuvant CRT MRI scan, the DWI sequence correctly identified CR in 28 cases based on the absence of bright signals on DWI on b-1000; 15 cases were false positive (FP) on post-CRT MRI. The reason for these false-positive cases on MRI could be the short time interval between post-CRT MRI scan acquisition and completion of neoadjuvant CRT, which led to misinterpretation of an area of high signal intensity at the location of the primary tumor as residual disease on post-CRT DWI images; additionally, some tumors had mucinous features, which also misled the interpretation. The accuracy of DWI in the interpretation of the tumor response is presented in the ROC curve, which shows an area under the curve (AUC) of 0.800. This means that the accuracy of post-CRT DWI in determining the presence or absence of tumors was 80%, which was statistically significant (p<0.0001) (Figure 1, Table 2).

**Table 2:** The accuracy of DWI in the ROC curve ^a^DeLong et al., 1988 [9]; ^b^binomial exact DWI: diffusion-weighted imaging; ROC: receiver operating characteristic

Variables	Values
Area under the ROC curve (AUC)	0.800
Standard error^a^	0.0378
95% confidence interval^b^	0.738 to 0.853
z statistic	7.937
Significance level p-value (area=0.5)	<0.0001

**Figure 2:**
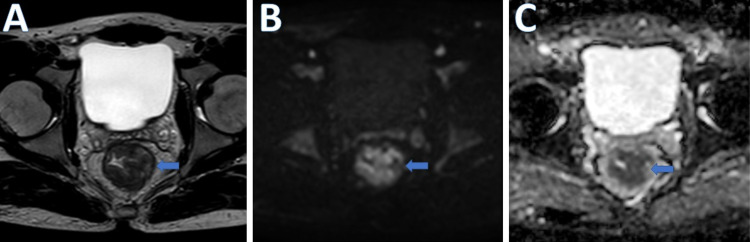
Pre-CRT images A. T2W MR axial image. B. DWI. C. ADC image of a 35-year-old man with pathologically proven moderately differentiated adenocarcinoma of the mid- and lower rectum (T3N1M0) showing circumferential tumor with diffusion restriction (blue arrows) CRT: chemoradiotherapy; DWI: diffusion-weighted imaging; ADC: apparent diffusion coefficient

**Figure 3:**
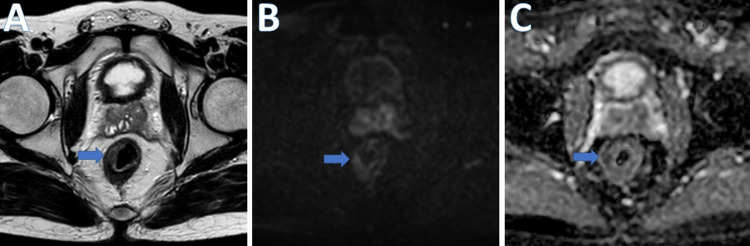
Post-CRT images A. T2W MR axial image. B. DWI. C. ADC image of a 35-year-old man with pathologically proven moderately differentiated adenocarcinoma of the mid- and lower rectum (T3N1M0) showing partial response with slight residual thickening and diffusion restriction at 9 O'clock position (blue arrows) CRT: chemoradiotherapy; DWI: diffusion-weighted imaging; ADC: apparent diffusion coefficient

Figure 2 and Figure 3 present the pre-CRT and post-CRT images respectively.

## Discussion

Rectal cancer is one of the most common malignancies of the gastrointestinal tract throughout the world, and it usually affects the elderly population who is above 50 years of age with a slightly higher incidence in males [10,11]. It has a high mortality rate due to the increased risk of recurrence and metastases [11]. Histologically, 98% of rectal cancers are adenocarcinoma [10]; therefore, this article primarily focused on rectal adenocarcinoma. These patients usually present with bleeding per rectum, painful defecation, and altered bowel habits [12].

Radiology plays a vital role in staging and restaging the rectal carcinoma and deciding on the next appropriate management step. The rectal tumors are usually staged and restaged on MRI. Recently, the use of DWI for restaging rectal cancer has been gaining popularity; therefore, in this study, we evaluated the role of DWI in post-CRT rectal cancers.

DWI is a sequence of MRI that measures the random Brownian motion of the water molecules within a tissue. Therefore, the changes in tissue cellularity and/or composition would affect the diffusion of the water molecules, which is then measured quantitatively using DWI. In clinical practice, DWI is most commonly acquired by the single-shot echo-planner imaging (SS-EPI) technique.

In our study, we observed that DWI is of great importance in the evaluation of post-treatment tumor response, i.e., a diagnostic accuracy of 91%. Similar results were also observed by Kim et al., i.e., 85 and 82% accuracy as observed by two different reviewers respectively [5]. The use of DWI to monitor ADC values in the advanced rectal cancer patients undergoing treatment was also observed by Kremser et al. [13] and Hein et al. [14]. We also observed in our study that the mean ADC values were significantly higher in patients who had a CR as compared to those who did not have CR. Kim et al. also noted a similar trend in the ADC values. Additionally, they also observed that ADC values in the mucinous tumors were also higher when compared to the adenocarcinomas. Hence, careful observation of the ADC values also plays an important role in the determination of CR in this histological category [5]. Furthermore, it is very difficult to anticipate the residual disease from inactive mucin in mucinous rectal cancers on conventional MR images [5,15]. Apart from the evaluation of tumor response in the primary tumor, DWI can also be used to evaluate the nodal metastases [1]. In their study, van Heeswijk et al. also advocated the supporting role of DWI in the selection of patients for organ preservation after CRT and suggested that the absence of lymph nodes in locally advanced rectal tumors after neoadjuvant CRT on restaging DWI can be a reliable predictor of negative nodal status [1].

Therefore, based on our findings, we believe that DWI plays a vital role in the evaluation of post-treatment tumor response. However, there are still some limitations in using this sequence. Firstly, the spatial resolution of DWI is limited, and the poor signal-to-noise ratio of high b-value images makes it impossible to identify the different layers of the rectal wall [16]. Secondly, DWI is imprecise in differentiating CR from near-CR as well as in the differentiation of the residual tumor from inactive mucin [5].

This study has some limitations, which need to be considered. Firstly, the design of the study we conducted was retrospective, and secondly, the number of patients was relatively small.

## Conclusions

The results of our study suggest that DWI is very useful in the evaluation of post-treatment tumor response with excellent diagnostic accuracy. Furthermore, DWI is also very valuable in the evaluation of metastatic nodes, and the absence of nodal disease on the DWI is a reliable predictor of negative nodal metastases. Therefore, DWI also aids in making an appropriate treatment plan and helps in the selection of patients for organ preservation after CRT.
